# Interstitial Lung Disease and Transverse Myelitis: A Possible Complication of COVID-19 Vaccine

**DOI:** 10.7759/cureus.21875

**Published:** 2022-02-03

**Authors:** Zunaira Khan, Ahmad Ammar Khattak, Nawal Rafiq, Anam Amin, Mahwish Abdullah

**Affiliations:** 1 Accident and Emergency, Kingston Hospital NHS Foundation Trust, London, GBR; 2 Pathology, Kabir Medical College, Gandhara University, Peshawar, PAK; 3 Accident and Emergency, Rehman Medical Institute, Peshawar, PAK; 4 Internal Medicine, Northwest General Hospital, Peshawar, PAK; 5 Anesthesia, Pakistan Air Force Hospital, Islamabad, PAK

**Keywords:** vaccine adverse events, covid 19 vaccine, acute transverse myelitis (atm), acute myelopathy, interstitial lung disease

## Abstract

The clinical impact of the severe acute respiratory syndrome 2 (SARS-CoV-2) pandemic is growing, and vaccine-associated complications are becoming more evident. Although global vaccination against coronavirus disease 19 (COVID-19) is an outstanding accomplishment, safety concerns and adverse outcomes are also emerging that need to be addressed promptly. The most reported side effects of the COVID-19 vaccine include fever, myalgia, headache, and injection site reactions. Acute transverse myelitis (ATM) and interstitial lung disease (ILD) following the CoronaVac vaccine are rarely reported. We report a case of ILD followed by acute myelopathy in a female who presented with dyspnea, cough, and fever after the second dose of the COVID-19 vaccine. On the third day of admission, she developed paresthesia and bilateral upper and lower limb weakness. She was diagnosed with ILD and ATM due to the COVID-19 vaccine based on imaging and detailed investigations after ruling out all possible causes. Her neurological and respiratory manifestations improved gradually after starting intravenous methylprednisolone.

## Introduction

Acute transverse myelitis (ATM) is an immune-mediated condition characterized by spinal cord inflammation, manifesting as autonomic, motor, or sensory deficits below the lesion level [1]. ATM can affect any spinal cord segment, with the cervical region being the most affected segment, followed by the thoracic region. ATM can be divided into non-compressive and compressive myelopathies, and non-compressive causes include idiopathic, infectious, post-vaccination, post-infectious, and paraneoplastic syndromes [2]. The most common etiology of ATM is idiopathic, and the infections causing ATM include human immunodeficiency virus, cytomegalovirus, herpes viruses, neuroborreliosis, mycoplasma, Zika Virus, and West Nile virus [3]. A meta-analysis reported 37 cases presenting signs and symptoms of ATM due to different vaccines, including hepatitis B, diphtheria, measles-mumps-rubella, polio, and other vaccines [4].

Interstitial lung disease (ILD) has a broad spectrum of clinical presentations ranging from transient lung inflammation to acute respiratory distress syndrome and manifests with ground-glass opacities with or without consolidation in peripheral, basal, or bilateral areas in multiple lobes [5]. ILD and ATM after vaccination are also underlined in the literature [6]. Even though ILD and ATM are sporadic disorders commonly associated with severe acute respiratory distress syndrome 2 (SARS-CoV-2), ILD and ATM due to coronavirus disease 19 (COVID-19) vaccination are rare, and a few cases have been reported [7,8]. We report a case of ILD and ATM caused by the COVID-19 vaccination in a single patient.

## Case presentation

A 61-year-old female with a past medical history of asthma and hypertension was admitted to the emergency department with complaints of dyspnea, cough, and fever for the last two days. She reported that her symptoms began after receiving the second dose of the CoronaVac vaccine two days ago. She was on the albuterol-beclomethasone inhaler for asthma and amlodipine for hypertension, and she reported compliance with her medications. She did not report any change in medications in the last six months. Initially, she had fever, nausea, and myalgia and took acetaminophen for symptomatic improvement. She had no allergies and denied smoking, alcohol abuse, and illicit drug use.

**Figure 1:**
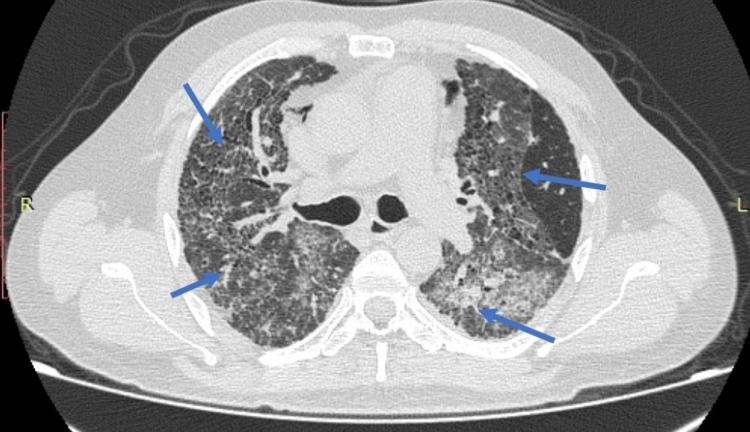
CT chest showing interstitial infiltrates in both lung fields. CT: Computed tomography

On initial evaluation, her vitals were stable except for tachypnea and low oxygen saturation. Chest X-ray revealed infiltrates in both lungs, and computed tomography (CT) of the chest demonstrated multiple asymmetrical ground-glass opacities in both lungs (Figure 1). The results of the initial laboratory analysis were normal except for elevated c-reactive protein (17.20 mg/dL), lactate dehydrogenase (302 U/L), and d-dimer (1.1 mg/L). Sputum cytology did not reveal any organism. Legionella pneumophilia antigen and pneumococcal antigen testing were negative. A quantitative test for COVID-19 antigen on admission was also negative. She was initially managed with supplemental oxygen. Her condition worsened gradually, for which she required mechanical ventilation. She was commenced on azithromycin and ampicillin/sulbactam based on the provisional diagnosis of pneumonia.

**Figure 2:**
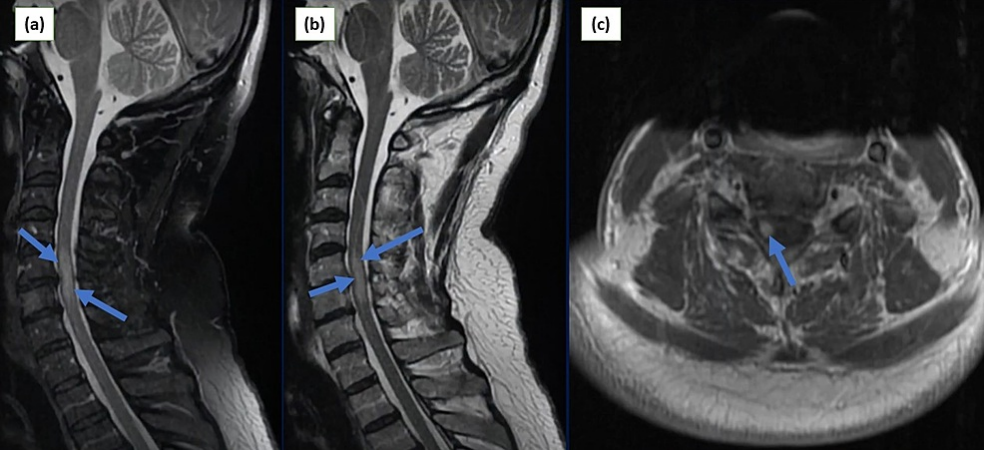
Cervical MRI images: (a) sagittal view demonstrating T1-weighted image with signal enhancement from C4-C6; (b) sagittal view showing T2-weighted image with hyperintense signals from C3-C5; (c) axial view of cervical spine demonstrating uniform enhancement at the level of C4. MRI: magnetic resonance imaging, C: cervical.

She reported improvements in her respiratory system, and she was extubated on the second day of admission. She reported sudden onset of abnormal sensations and weakness in bilateral upper and lower limbs on the third day. Neurological examination revealed upper motor neuron disease signs, including hyperreflexia, hypertonia, and spasticity, in both upper and lower extremities with increased severity in legs. She also had hypoesthesia in the upper and lower limbs. Cranial nerves were intact, and there were no signs of meningeal irritation. She denied diplopia, facial weakness, neck stiffness, dysarthria, or dyspnea. Magnetic resonance imaging (MRI) of the whole spine was performed, which showed an expansile central hyperintense signal in the cervical segment of the spinal cord (Figure 2). Brain MRI showed age-related changes. Cerebrospinal fluid (CSF) analysis revealed no abnormality (Table 1).

**Table 1:** The result of CSF analysis. RBC: red blood cell, LDH: lactate dehydrogenase, CSF: cerebrospinal fluid.

CSF analysis	Lab result	Reference
RBC	Not detected	< 1/mm^3^
Nucleated cell	2	< 8/mm^3^
Protein	40	< 45 mg/dl
Glucose	67	40-70 mg/dl
Color	Colorless	Colorless
Appearance	Clear	Clear
LDH	21	< 40 U/L

The results of autoimmune screening, including antinuclear antibodies, rheumatoid factor, anticardiolipin, and atypical antibodies, including aquaporin-4 antibodies (AQP4-ab), were negative. Infectious workup for human immunodeficiency virus, herpesvirus, varicella-zoster virus, and syphilis serology was also negative. The findings were suggestive of transverse myelitis.

She was started on intravenous methylprednisolone 1g/kg/day along with the current management, and physical therapy was initiated. She reported improvement in sensation in the affected limbs. She was discharged to a rehabilitation facility with aggressive physical and occupational therapy. At her recent follow-up two weeks later, she had started walking with a cane, and a significant improvement in her condition was noted. She is currently on low-dose methylprednisolone and improving well with no further exacerbations.

## Discussion

Despite the excellence of this achievement amid the pandemic, post-marketing surveillance and adverse outcomes associated with the COVID-19 vaccines are still required [6,9]. Reports of COVID-19 vaccine-associated adverse effects emerged after the vaccines became widely available [10]. Significant complications related to the COVID-19 vaccine include seizures, stroke, acute disseminated encephalomyelitis, pericarditis, pancreatitis, acute respiratory distress syndrome [11]. The most-reported neurological side effects of the COVID-19 vaccine include headache, dizziness, myalgia, and paresthesia [12]. A small number of ATM cases caused by COVID-19 vaccination have also been underlined [13,14]. Tahir et al. reported a case of ATM after the COVID-19 vaccine, and the latency period between vaccination and onset of symptoms was 10 days [13]. Similarly, Khan et al. also highlighted an ATM case after Moderna COVID-19 vaccination [14]. Likewise, cases of ILD after COVID-19 vaccines have also been reported [15]. Yoshifuji et al. described a case of ILD after vaccination [15]. Kono et al. also reported a case of ILD after two weeks of COVID-19 immunization [16].

COVID-19 and influenza virus vaccines are commonly linked to ILD and ATM, and many sporadic cases of ILD and ATM due to vaccinations have also been reported [10,17]. Pathophysiology of vaccine-induced complications is molecular mimicry, and vaccine adjuvants can induce autoimmunity similar to infectious agents [5]. An acute autoimmune reaction occurs in molecular mimicry due to cross-reactivity with structurally related host proteins [6]. Hyperinflammatory response, cytokine upregulation, epitope spreading, and activation of B and T cells due to the COVID-19 vaccine can lead to the occurrence of neurological and respiratory manifestations by stimulating immune reactions [18]. A recently published article underlined the immunological reactions between COVID-19 spike protein antibodies and host proteins and their interactions with myelin basic protein that might contribute to the pathogenesis of the demyelinating autoimmune disease [19,20]. Furthermore, angiotensin-converting enzyme 2 (ACE2) receptors are expressed in the respiratory tract, gastrointestinal, and nervous systems, particularly in the blood-brain barrier. Their interaction with the viral spike proteins can also trigger the hyperinflammatory response [20].

In our case, the patient was diagnosed with ILD and ATM. Though she had bronchial asthma and hypertension, she had been stable for several years and had no drug reactions or underlying pulmonary and neurological disease. Therefore, we consider the COVID-19 vaccine could trigger ILD and ATM. Additionally, her symptoms improved gradually after commencing methylprednisolone. We assume that the chronological association of vaccines with the development of ATM, and ILD, the absence of any infectious etiology, and the effective clinical response to steroids support the notion that the COVID-19 vaccine is the etiology of both ILD and ATM.

## Conclusions

To our knowledge, we present the first case of ILD and ATM due to the COVID-19 vaccine in a single patient, and our case highlights the association of the COVID-19 vaccine with the development of ILD and ATM. Diagnosis requires a high index of suspicion in patients manifesting atypical symptoms after COVID-19 vaccination. Our case also warrants further study to establish the causality and association between the COVID-19 vaccine and the occurrence of vaccine complications, including ATM and ILD.
